# Multi-omics profiling reveals epidermal growth factor as a potential biomarker and therapeutic target in lupus nephritis and ANCA-associated vasculitis with rapidly progressive glomerulonephritis

**DOI:** 10.1371/journal.pone.0349307

**Published:** 2026-05-29

**Authors:** Mengli Xu, Qi Cheng, Yifan Xie, Xin Chen, Lingjiang Zhu, Wenjing Su, Yan Du, Huaxiang Wu, Keda Lu

**Affiliations:** 1 Department of Nephrology, The Third Affiliated Hospital of Zhejiang Chinese Medical University, Hangzhou, China; 2 Department of Rheumatology, The Second Affiliated Hospital of Zhejiang University School of Medicine, Hangzhou, China; 3 Department of Rheumatology, Jinhua Municipal Central Hospital, Jinhua, China; 4 Department of Nephrology, Shandong Provincial Hospital affiliated to Shandong First Medical University, Jinan, China‌‌; Kitasato University, JAPAN

## Abstract

The kidney is a primary target organ in systemic lupus erythematosus (SLE) and ANCA-associated vasculitis (AAV), which frequently manifest as lupus nephritis (LN) and renal AAV, respectively. A severe acute presentation of both diseases is rapidly progressive glomerulonephritis (RPGN), leading to rapid loss of renal function. However, the shared molecular mechanisms underlying this aggressive phenotype remain poorly defined. This study aimed to identify key biomarkers and pathological processes in acute autoimmune-related glomerulonephritis, with a focus on LN and AAV presenting as RPGN. Through comparative transcriptomic analysis of glomerular samples from patients with LN, AAV, and RPGN against healthy controls, we identified a total of 180 common differentially expressed genes, comprising 33 upregulated and 147 downregulated genes. Pathway analysis revealed enhanced immune and inflammatory responses, including neutrophil extracellular trap (NET) formation, accompanied by significant impairments in amino acid and fatty acid metabolism. A core set of 12 hub genes was identified, and network analysis suggested specific transcriptional and post-transcriptional regulatory axes. Immune infiltration analysis demonstrated a common pattern characterized by decreased resting immune cells and increased activated cells—including memory CD4 ⁺ T cells, NK cells, and mast cells—along with elevated monocyte levels, particularly in LN. Notably, epidermal growth factor (EGF) was significantly downregulated and strongly correlated with impaired renal function, demonstrating good diagnostic performance. These findings suggest that EGF may serve as a potential biomarker for LN and RPGN. This study provides the first systematic transcriptomic analysis of LN and AAV presenting as RPGN, highlighting enhanced inflammation (including NETosis) and dysregulated amino acid/fatty acid metabolism as key glomerular pathological features. Targeting EGF signaling and monocyte differentiation may offer novel therapeutic strategy for LN and AAV presenting as RPGN.

## Introduction

The kidney is the most commonly affected organ in autoimmune diseases characterized by vasculitis, such as systemic lupus erythematosus (SLE) and ANCA-associated vasculitis (AAV). Renal involvement manifests in at least half of SLE patients as lupus nephritis (LN) and in nearly 70% of AAV patients, significantly accelerating disease progression [1,2]. A substantial proportion (10–30%) of these patients progress to end-stage renal disease [3]. The considerable heterogeneity in clinical presentation and pathogenesis complicates diagnosis and treatment for rheumatologists. Therefore, early and accurate detection of renal involvement, enabling timely intervention, is crucial for improving prognoses in SLE and AAV.

The pathological spectrum of LN is highly diverse, encompassing lesions such as membranoproliferative glomerulonephritis, membranous nephropathy, podocyte injury, crescent formation, and sclerotic glomerulonephritis [4,5]. While most LN research focuses on the pathogenesis and long-term management of chronic kidney involvement, acute-phase manifestations, particularly rapidly progressive glomerulonephritis (RPGN), remain relatively unexplored [6]. RPGN, a severe complication leading to progressive renal deterioration and rapid acute renal failure without prompt intervention, is a hallmark of AAV and also occurs in LN. Current understanding of autoimmune-related glomerulonephritis, especially when LN presents as RPGN, remains limited. Pathologically classified as ISN/RPS class III or IV, RPGN affects approximately 5% to 15% of LN patients [7,8]. These cases are often managed using protocols derived from AAV, yet outcomes remain poor. Consequently, elucidating the pathogenesis of RPGN in both LN and AAV holds substantial clinical significance.

Although LN and AAV are fundamentally distinct in their pathogenesis—immune complex-mediated injury in LN versus pauci-immune necrotizing inflammation in AAV—both can present as rapidly progressive glomerulonephritis (RPGN) with crescent formation and rapid loss of renal function. This shared severe clinical phenotype suggests that despite differing initial triggers, common downstream pathways of acute glomerular injury may exist. Understanding these convergent mechanisms could identify biomarkers and therapeutic targets applicable across autoimmune RPGN. Therefore, this study focuses on LN and AAV as representative autoimmune diseases that manifest as RPGN, aiming to uncover shared molecular signatures rather than to equate their etiologies.

In this study, we analyzed transcriptomic data from patients with LN, AAV, and RPGN to identify biomarkers for acute autoimmune-related glomerulonephritis and evaluate their diagnostic, therapeutic, and prognostic potential. We further employed enrichment analysis, molecular interaction network construction, and immune infiltration assessment to delineate the regulatory mechanisms of hub genes during the acute phase. Our findings aim to clarify the pathogenesis of RPGN in LN and AAV, ultimately informing targeted therapeutic strategies.

## Materials and methods

### Patient samples and informed consent

Renal biopsy tissues were obtained from eight clinically diagnosed SLE patients with LN and five non-tumor renal tissues from nephrectomy specimens at the Second Affiliated Hospital of Zhejiang University School of Medicine (25/06/2020–31/10/2021). The study was approved by the Ethics Committee of the Second Affiliated Hospital of Zhejiang University School of Medicine, Hangzhou, China (approval number: 2020−306). All SLE patients met the American College of Rheumatology 1997 criteria and the Systemic Lupus International Collaborating Clinics 2012 criteria for SLE [9,10]. LN was classified according to the 2003 International Society of Nephrology/Renal Pathology Society (ISN/RPS) consensus [9]. The clinical characteristics of the 8 LN patients and 5 living donors (LDs) are shown in S1 Table (All the lab data from urine and serum were obtained at the time of kidney biopsy).

### Immunohistochemistry

Immunohistochemical staining was performed as previously described [10]. The primary antibody used was anti-EGF (27141–1-AP, Proteintech).

### Acquisition of mRNA microarray expression data

We searched the Gene Expression Omnibus (GEO) database for mRNA microarray expression datasets related to LN, AAV, and RPGN. Selection criteria included: (1) renal biopsy tissues from LN, AAV, RPGN patients and living donors (LDs), and (2) datasets containing >5 samples from both healthy controls and patients. Three datasets meeting these criteria were downloaded: two test sets, GSE104948 (21 LD, 22 RPGN [vasculitis], and 32 LN glomerular samples) and GSE108109 (6 LD and 15 AAV glomerular samples), and one validation set, GSE32591 (14 LD and 32 LN glomerular samples).

### Data normalization and identification of differentially expressed genes

Raw data were downloaded from GEO. The affy package in R software (version 4.0.1) was used for data preprocessing and normalization using the Robust Multiarray Average (RMA) method [11]. Differential gene expression analysis was conducted using the Limma package, with multiple hypothesis testing correction via the false discovery rate (FDR). Adjusted P-values < 0.05 and |log2(fold change)| > 1 were considered statistically significant for DEG identification [12,13]. For the purpose of this study, “common DEGs” were defined as genes that were significantly differentially expressed in the same direction (all upregulated or all downregulated) across all three conditions (RPGN, LN, and AAV) compared with healthy controls.

### Enrichment analysis

Gene Ontology (GO) and Kyoto Encyclopedia of Genes and Genomes (KEGG) pathway enrichment analyses were performed using DAVID (https://david.ncifcrf.gov/home.jsp) and WebGestalt (http://www.webgestalt.org/) online tools [14,15], the results of which were intersected with the next analysis. A Q value < 0.05 (adjusted P-value) indicated significant enrichment. Pathway analysis for miRNAs was conducted using DIANA-miRPath v3.0 (http://www.microrna.gr/miRPathv3).

### Construction of the PPI network and transcription factor (TF)-miRNA-mRNA regulatory network

The STRING database https://string-db.org/) was used to construct a Protein-Protein Interaction (PPI) network, visualized with Cytoscape (v3.8.0). The MCODE plugin identified significant network clusters (settings: degree cutoff = 2, node score cutoff = 0.2, K-core = 2, max depth = 100). Hub genes were identified by intersecting results from 12 algorithms within the cytoHubba plugin, visualized using the R package “UpSetR” [16]. MiRNAs regulated hub genes were predicted by four online miRNA databases including DIANA-microT v5.0 (http://www.microrna.gr/webServer), miRWalk (http://mirwalk.umm.uni-heidelberg.de/), and miRcode (http://www.mircode.org/index.php). TF-regulated miRNAs and mRNAs were predicted by TransmiR v2.0 (http://www.cuilab.cn/transmir) and ORTI (http://orti.sydney.edu.au), respectively. Regulatory networks were constructed and visualized in Cytoscape. The ClueGO + CluePedia plugin was used for KEGG pathway analysis of network components.

### Immune infiltration analysis

The relative abundances of 22 immune cell types in glomerular samples from LN, AAV, and RPGN datasets were estimated using the CIBERSORT algorithm implemented in R.

### Analysis of the relationship between EGF expression and clinical features of LN and RPGN based on the Nephroseq database

Clinical data and EGF expression for LN and RPGN (GSE104948 cohort) were analyzed using the Nephroseq (https://nephroseq.org/).

### Statistics analysis

Data analysis and visualization were performed using R software (v4.0.1) and GraphPad Prism 9. Differences between groups were assessed using an unpaired two-tailed Student’s t-test or the Mann-Whitney U test, as appropriate. Receiver operating characteristic (ROC) curves were plotted, and the area under the curve (AUC) was calculated using IBM SPSS Statistics 25. Correlations between variables were evaluated using Spearman’s rank correlation analysis via the R package “corrplot.” A P-value < 0.05 was considered statistically significant.

## Results

### Identification of DEGs in glomerulus compartment of RPGN, LN, and AAV

**Fig 1:**
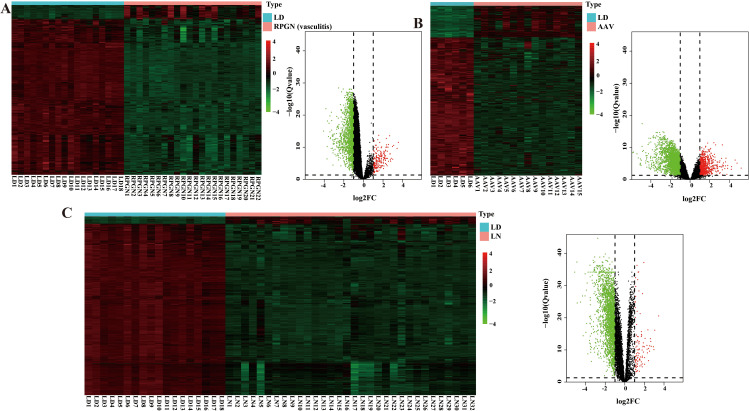
Identification of ‌‌DEGs. ‌A. Heatmap and volcano plot of DEGs between the RPGN samples and the LD samples. B. Heatmap and volcano plot of DEGs between the AAV samples and the LD samples. C. Heatmap and volcano plot of DEGs between the LN samples and the LD samples. RPGN, rapidly progressive glomerulonephritis; AAV, ANCA-associated vasculitis LN, lupus nephritis; LD, living donors. Values of *P* < 0.05 were considered significant.

Compared to healthy controls, we identified 2,289 DEGs in RPGN (192 upregulated, 2,097 downregulated), 2,559 DEGs in LN (107 upregulated, 2,452 downregulated), and 3,657 DEGs in AAV (683 upregulated, 2,974 downregulated) glomeruli. Heatmaps and volcano plots visualizing these DEGs are shown in Fig 1A-C.

### Enrichment analysis of common DEGs in glomerular compartments of RPGN, LN, and AAV

**Fig 2:**
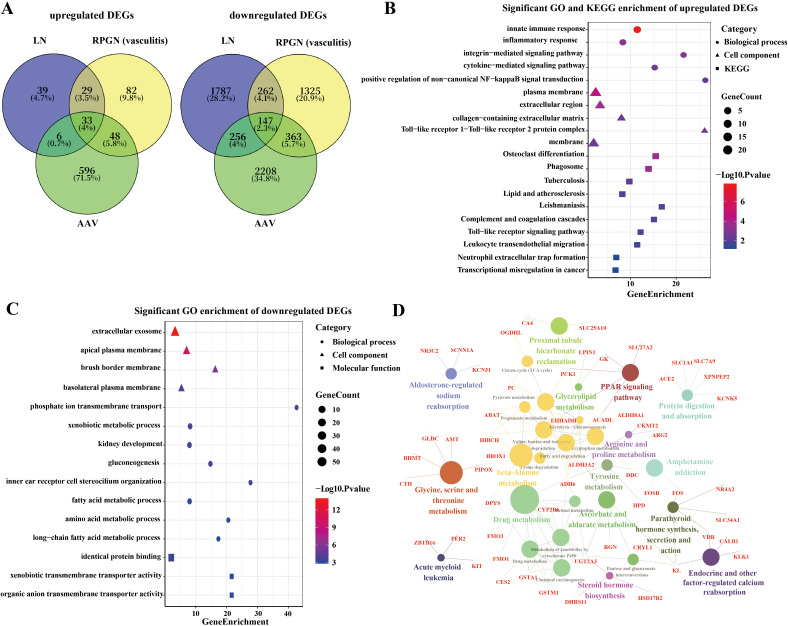
GO and KEGG pathway enrichment analyses of DEGs. A. Venn diagram showed the intersection of DEGs in the glomerular compartment of RPGN, LN and AAV. B. The bubble plot showing the most enriched GO terms and KEGG pathways of upregualted DEGs. C. The bubble plot showing the most enriched GO terms of downregualted DEGs. D. The network plot showing the most enriched GO terms of downregualted DEGs. RPGN, rapidly progressive glomerulonephritis; AAV, ANCA-associated vasculitis LN, lupus nephritis; LD, liver donors; BP, biological process; CC, cell component. Values of *P* < 0.05 were considered significant.

Intersecting DEGs from the three conditions revealed 33 commonly upregulated and 147 commonly downregulated genes (Fig 2A, B). GO and KEGG analysis indicated that upregulated genes were primarily enriched in immune and inflammatory responses, including cytokine-mediated signaling, integrin binding, toll-like receptor signaling, and neutrophil extracellular trap (NET) formation (Fig 2C). Downregulated genes were associated with extracellular exosome function, kidney development, transmembrane transport, and amino/fatty acid metabolic processes (Fig 2D). KEGG analysis further highlighted impairments in amino acid/fatty acid metabolism, renal reabsorption, and hormone synthesis/metabolism during the acute phase of autoimmune-related glomerulonephritis (Fig 2E).

### Construction of PPI and TF-miRNA-mRNA regulatory networks

**Fig 3:**
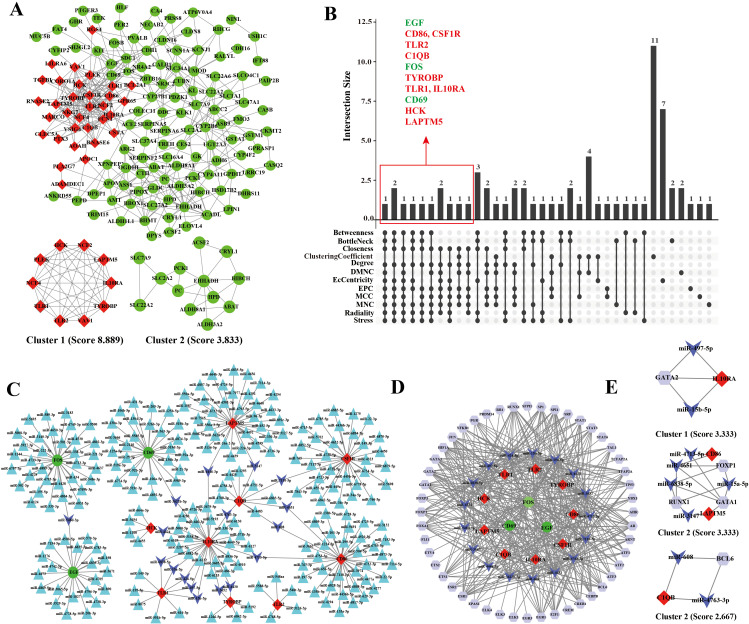
Construction of protein-protein interaction network and TF-miRNA-mRNA regulatory network. A. The interaction network between proteins coded by DEGs. B. The upset plot of 12 cytohubba algorithms. C. The miRNA-mRNA regulatory network included 240 miRNAs and 12 mRNAs. D. The TF-miRNA-mRNA regulatory network included 49 TFs, 17 miRNAs and 12 mRNAs. E. Three cluster modules extracted by MCODE.

A PPI network of common DEGs contained 180 nodes and 424 edges. MCODE identified two key clusters: Cluster 1 (score: 8.889) contained 10 upregulated genes, and Cluster 2 (score: 3.833) contained 13 downregulated genes (Fig 3A). Intersection analysis identified 12 hub genes: three downregulated (EGF, FOS, CD69) and nine upregulated (CD86, CSF1R, C1QB, TLR1, TLR2, TYROBP, IL10RA, HCK, LAPTM5) (Fig 3B). A regulatory network involving 240 miRNAs and these 12 hub genes was constructed (Fig 3C). CD86 was targeted by the most miRNAs (n = 37), while 17 miRNAs each regulated two hub genes. These 17 miRNAs were enriched in pathways related to fatty acid biosynthesis/metabolism, hormone biosynthesis, and drug metabolism, partially overlapping with DEG enrichment patterns (S1 Fig). Integration with TF predictions yielded a comprehensive 49 TFs-17 miRNAs-12 mRNAs regulatory network (Fig 3D) with three transcriptional cluster modules (Fig 3E).

### Pattern of immune cell infiltration in glomerular compartments of RPGN, LN, and AAV

**Fig 4:**
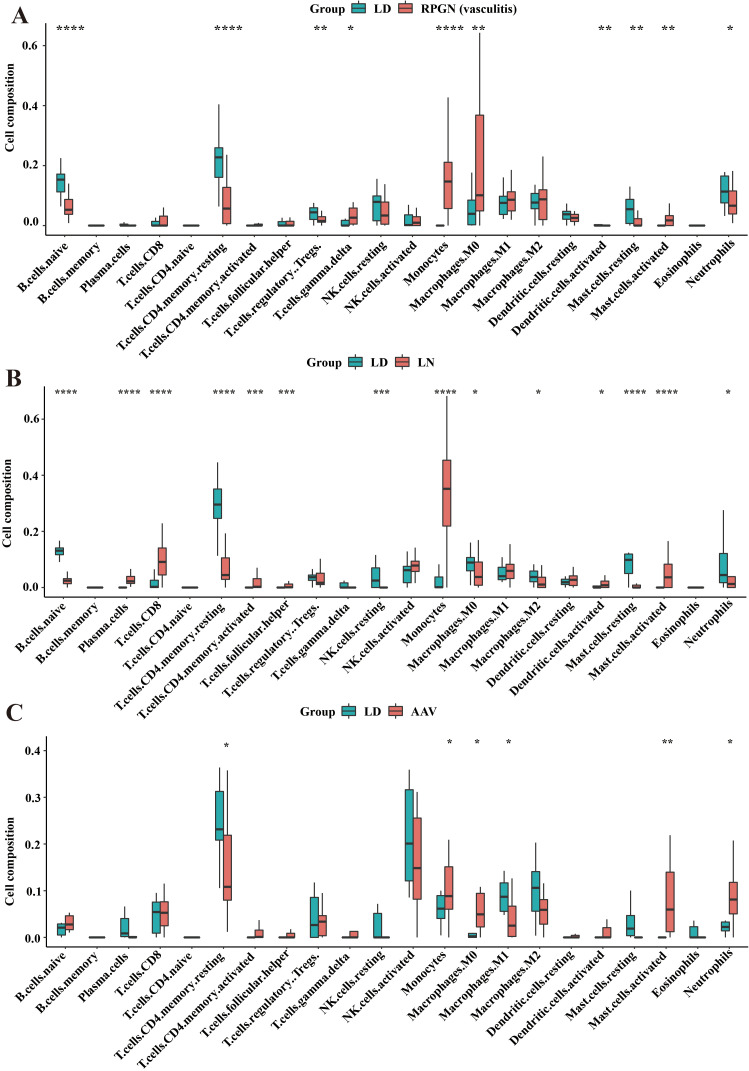
Pattern of Immune Cell Infiltration in Glomerular Compartments of RPGN, LN, and AAV. A-C. The levels of 22 types of immune cells in live donors and RPGN (A), LN (B), AAV (C) patients. *, *P* < 0.05; **, *P* < 0.01; ***, *P* < 0.001; ****, *P* < 0.0001. *P* < 0.05 was considered statistically significant.

**Fig 5:**
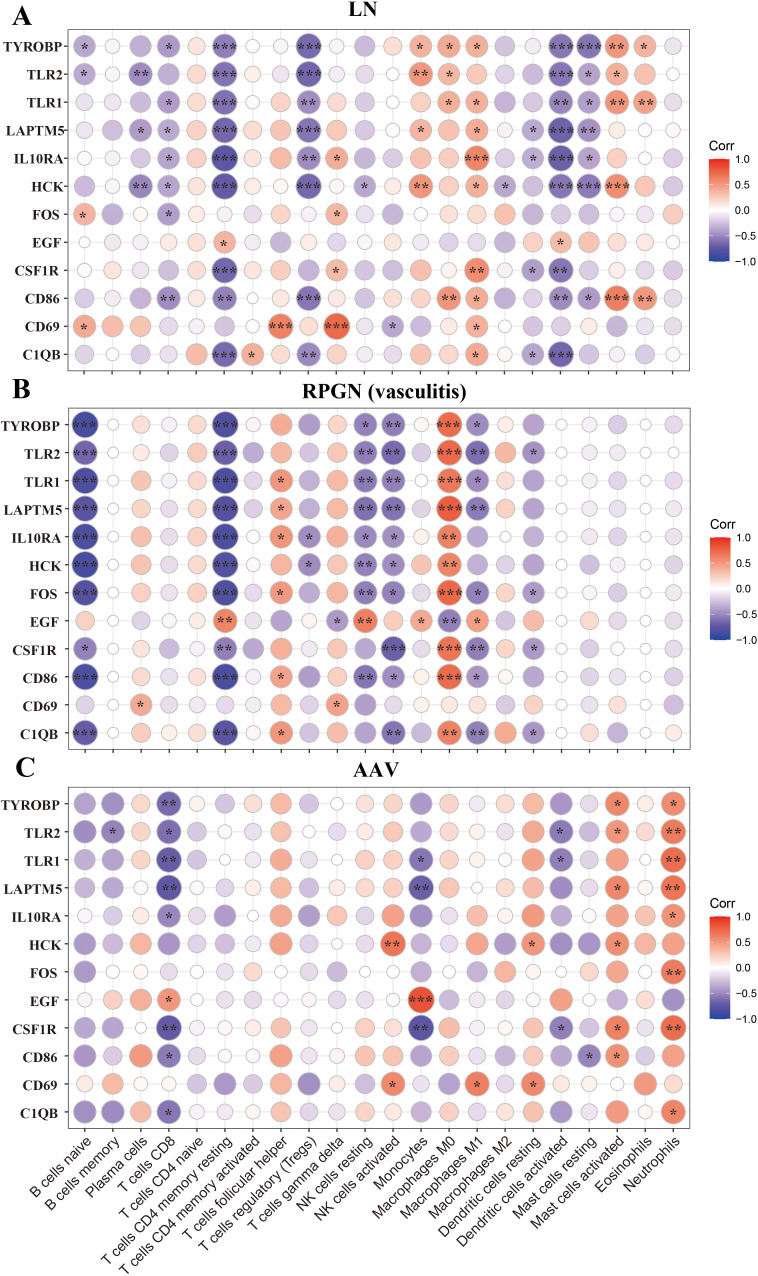
The correlation between expression of 12 hub genes and the level of infiltrating immune cells. A-C. The correlation between expression of 12 hub genes and levels of 22 types of immune cells in RPGN (A), LN (B), AAV (C) patients. *, *P* < 0.05; **, *P* < 0.01; ***, *P* < 0.001; ****, *P* < 0.0001. *P* < 0.05 was considered statistically significant.

Acute autoimmune glomerulonephritis shared a common immune infiltration pattern characterized by decreased resting immune cells and increased activated cells (memory CD4 ⁺ T cells, NK cells, mast cells), alongside elevated monocyte levels—particularly prominent in LN. Disease-specific variations were observed in B cells, neutrophils, and macrophage polarization. LN exhibited more pronounced immune activation than RPGN or AAV, with significant increases in plasma cells, follicular helper T cells, CD8 ⁺ T cells, and activated dendritic cells (Fig 4A-C). Correlation analysis in LN revealed that most upregulated hub genes correlated negatively with resting CD4 ⁺ memory T cells, mast cells, regulatory T cells, and activated dendritic cells, but positively with monocytes and M1 macrophages. In RPGN, upregulated hub genes correlated negatively with resting CD4 ⁺ memory T cells, NK cells, and dendritic cells, but positively with follicular helper T cells and M0 macrophages. In AAV, positive correlations were observed with neutrophils and activated macrophages, while negative correlations were seen with CD8 ⁺ T cells (Fig 5A-C).

### Relationship between EGF and clinical characteristics in LN and RPGN

**Fig 6:**
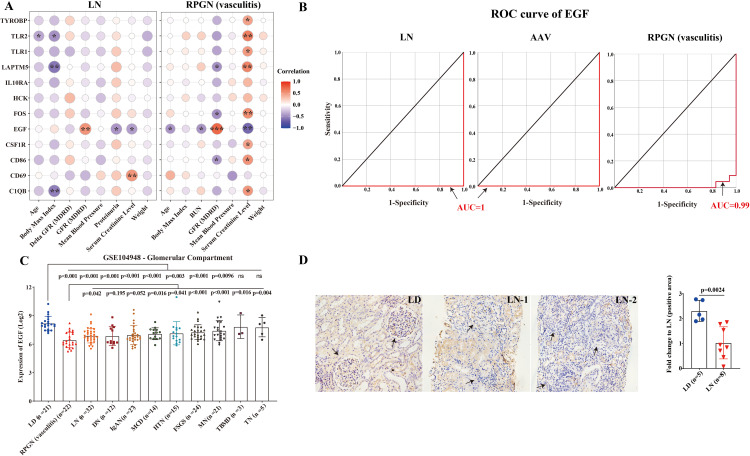
Relationship between hub genes and clinical characteristics in LN and RPGN. A. The correlation between expression of 12 hub genes and clinical characteristics in LN and RPGN. B. ROC curve of EGF in LN, AAV and RPGN (vasculitis). C. Expression of EGF within the glomerular compartments of various renal diseases and living donors in GSE104948 cohort. D. Representative immunohistochemistry images of EGF in LN patients and LDs. In the patient 1 with LN, no crescents were observed in the glomeruli. In the patient 2 with LN, crescents were present in the glomeruli. The black arrows indicate the glomerulus. LD, living donors; LN, lupus nephritis; RPGN, rapidly progressive glomerulonephritis; DN, diabetic nephropathy; HTN, hypertensive nephropathy; IgAN, IgA nephropathy; FSGS, focal segmental glomerular sclerosis; TMD, thin membrane disease; MCD, minimal change disease; MGN, membranous glomerulonephropathy; TBMD, thin basement membrane nephropathy; TN, tumor nephrectomy. *, *P* < 0.05; **, *P* < 0.01; ***, *P* < 0.001. *P* < 0.05 was considered statistically significant.

Among these 12 hub genes, only EGF expression correlated positively with serum creatinine, blood urea nitrogen, and proteinuria levels, and negatively with glomerular filtration rate (GFR) in both LN and RPGN (vasculitis). EGF expression was lower in active class III/IV LN but showed no association with gender, age, or body mass index (Figs 6A and S2). EGF demonstrated high diagnostic value, with AUC values exceeding 0.99 in ROC analyses for LN, AAV, and RPGN (vasculitis) (Figs 6B and S3). Notably, EGF expression in RPGN (vasculitis) glomeruli was significantly lower than in other nephropathies, including hypertensive kidney disease, FSGS, IgAN, MCD, and MGN (Fig 6C). This downregulation was confirmed by validation set (GSE32591 cohort) and immunohistochemistry in human LN tissues (Figs 6D and S4). Importantly, EGF expression was further reduced in glomeruli with crescent formation (LN patient 2) compared to those without (LN patient 1) (Fig 6D).

## Discussion

Assessment of LN severity integrates clinical and pathological parameters. Current treatment strategies, often guided by high-quality randomized controlled trials, prioritize pathological classification. However, LN presenting as RPGN poses a distinct clinical challenge due to its rapid progression, relatively low prevalence, and pronounced heterogeneity, which have limited robust comparative studies. Although LN and AAV are etiologically distinct, with immune complex deposition and complement activation characterizing LN, while AAV typically exhibits pauci-immune necrotizing glomerulonephritis. However, both can lead to RPGN. This clinical overlap raises the possibility of shared downstream molecular mechanisms driving acute glomerular injury in autoimmune-related RPGN. To address this knowledge gap, the present study performed a bioinformatic analysis to identify such convergent pathways and provide mechanistic insights that may inform future therapeutic strategies.

Consistent with previous reports, we identified numerous DEGs in RPGN, LN, and AAV glomeruli. Enrichment analysis confirmed that enhanced immune/inflammatory responses and impaired amino acid/fatty acid metabolism are pivotal alterations in acute autoimmune-related glomerulonephritis. In RPGN, a robust inflammatory response induces severe glomerular injury and subsequent rapid functional decline, which may underlie the observed disruption of amino acid transport, fatty acid reabsorption, and β-oxidation [17]. Notably, fatty acid oxidation (FAO) and uptake regulate M2 macrophage polarization, while stable amino acid transporter expression promotes M1 polarization [18–21]. Therapeutic agents targeting FAO, such as rosiglitazone (a PPAR-γ agonist) and fish oil, promote M2 differentiation and ameliorate LN [22]. Our immune infiltration analysis revealed elevated monocytes in all three conditions and reduced M2 macrophages in LN and AAV, suggesting that M1/M2 imbalance exacerbates renal injury during RPGN progression. Modulating monocyte differentiation thus represents a potential therapeutic avenue.

Another key finding of our study is the consistent enrichment of NETosis-related pathways in both LN and AAV presenting as RPGN. NETs are well-documented drivers of vascular inflammation in AAV, serving as sources of autoantigens (e.g., MPO, PR3) and activators of the alternative complement pathway [23,24]. In LN, accumulating evidence indicates that NETs contribute to immune complex deposition, type I interferon production, vascular injury, tissue inflammation and fibrosis [25–27]. The shared upregulation of NETosis in our transcriptomic analysis suggests that this process may represent a convergent pathogenic mechanism underlying crescent formation and rapid renal function decline, irrespective of the initial immune-complex or pauci-immune nature of the disease. This observation supports the rationale for comparing these two diseases in the context of RPGN and highlights NETosis as a potential therapeutic target.

PPI network analysis identified 12 hub genes. Eleven of these (all except FOS) showed high diagnostic value as candidate biomarkers for LN and AAV presenting as RPGN. Regulatory network analysis implicated specific axes in pathogenesis, including GATA2 → miR-497-5p/miR-15b-5p → IL10RA; BCL6 → miR-4763-3p/miR-608 → C1QB; FOXP1/GATA1/RUNX1 → miR-4651 → CD86; and RUNX1 → miR-3147 → LAPTM5. Published evidence links CD86, IL10RA, C1QB, and LAPTM5 to crescent formation via macrophage polarization, Th17/Treg balance, and complement activation, warranting functional validation [28–31]. LN exhibited heightened adaptive immunity, consistent with antibody deposition and immune complex accumulation [32,33]. Crucially, innate immune cells—monocytes, macrophages, mast cells, and NK cells—showed marked alterations across all conditions, initiating and sustaining inflammatory injury. Elucidating innate immune mechanisms and intercellular crosstalk will reveal new immunomodulatory targets.

Epidermal Growth Factor (EGF), specifically expressed in the kidney (S5 Fig), is synthesized in glomeruli, Henle’s loop, and distal tubules. Previous studies report that reduced urinary EGF correlates with histological damage and poor renal outcomes in LN and AAV [34–36]. Our data confirm that EGF downregulation associates with worsened renal function (elevated creatinine, proteinuria, reduced GFR) in LN and RPGN, supporting its diagnostic utility. EGF expression also correlated with specific immune cells, suggesting microenvironmental crosstalk. Studies in diabetic nephropathy models and ischemic acute renal failure have demonstrated protective effects of EGF through modulation of autophagy and promotion of podocyte and tubule cell regeneration [37,38]. Moreover, genetic evidence from EGF-deficient mice reveals that EGF deficiency can lead to severe crescentic glomerulonephritis [39]. These findings not only reinforce its essential role in maintaining renal homeostasis but also support the diagnostic and therapeutic potential of EGF.

This study has several limitations. First, the immune infiltration patterns were derived from computational deconvolution (CIBERSORT) without experimental validation through gold-standard methods like flow cytometry. Second, while hub gene expression was validated bioinformatically and via IHC, their diagnostic utility remains to be established in independent prospective cohorts, and functional studies are needed to confirm their pathogenic roles. Third, the human sample size was limited, and critical AAV patient details, including ANCA subtypes, disease activity scores, and medication histories, were incomplete, preventing adjustment for potential confounders. Fourth, the predicted regulatory networks (TF-miRNA-mRNA) and hypothesized links between metabolic dysregulation and immune polarization remain purely computational, requiring experimental validation through ChIP-qPCR, luciferase assays, and mechanistic studies. Finally, more basic experiments and prospective cohort studies are required to validate the therapeutic potential of EGF and NETs inhibition or monocyte differentiation modulation.

In conclusion, this study provides the first systematic transcriptomic analysis of LN and AAV manifesting as RPGN. We identified enhanced inflammation, NETs and disrupted amino acid/fatty acid metabolism as central pathological features in glomerular compartments. Our findings nominate EGF signaling and monocyte differentiation pathways as potential therapeutic targets for autoimmune-related glomerulonephritis presenting as RPGN. Future studies incorporating other immune complex-mediated diseases (e.g., IgA nephropathy) and experimental validation will further clarify the specificity and generalizability of our observations.

## Supporting information

S1 Table Clinical characteristics of the 8 LN patients and 5 LDs.
(DOCX)

S1 Fig Pathway enrichment of 17 miRNAs by DIANA-miRPath v3. 0 tool.
(TIF)

S2 Fig Relationship between hub genes and clinical characteristics in LN.
A. Expression of EGF within the glomerular compartments in different classes of LN patients from GSE104948 cohort. B. Expression of EGF within the glomerular compartments in female and male LN patients from GSE104948 cohort. C-D. The correlation between expression of EGF and serum creatinine level (C), glomerular filtration rate (GFR) (D), proteinuria level (E), body mass index (F) and age (H) in LN patients. *, *P* < 0.05; **, *P* < 0.01; ***, *P* < 0.001; ns, no significant. *P* < 0.05 was considered statistically significant.
(TIF)

S3 Fig ROC curve of 12 hub genes in LN, AAV and RPGN (vasculitis).
(TIF)

S4 Fig Expression of EGF in validation set (GSE32591 cohort).
(TIF)

S5 Fig Expression of EGF in in different organs or tissues (BioGPS).
(TIF)

## Abbreviations

LN: lupus nephritis
AAV: ANCA-associated vasculitis
SLE: systemic lupus erythematosus
LD: living donors
RPGN: rapidly progressive glomerulonephritis
GEO: Gene Expression Omnibus
DEG: differentially expressed gene
TF: transcription factor
HTN: hypertensive kidney disease
FSGS: Focal segmental glomerular sclerosis
IgAN: IgA nephropathy
MCD: minimal change disease
MGN: membranous glomerulonephropathy
GFR: glomerular filtration rate
NET: neutrophil extracellular trap
