# Supplementary material for: Multi-omics profiling reveals epidermal growth factor as a potential biomarker and therapeutic target in lupus nephritis and ANCA-associated vasculitis with rapidly progressive glomerulonephritis
Source: PLoS One. 2026 May 29;21(5):e0349307. doi: 10.1371/journal.pone.0349307 (PMC13221000; doi:10.1371/journal.pone.0349307)
Supplement: S1 Table — Clinical characteristics of the 8 LN patients and 5 LDs. (DOCX) [file pone.0349307.s001.DOCX]

**Supplementary Table 1 Clinical characteristics of the 8 LN patients and 5 LDs**

| Items | | **Patients with LN (N=8)** | **Living donors (N=5)** |
| --- | --- | --- | --- |
| **General features** | |  |  |
| Age (Years), mean±SD | | 31.38±7.11 | 33.25±6.35 |
| Sex (Female/male) | | 7/1 | 5/0 |
| Course (Years), median (range) | | 3.5 (0.04-13) | N/A |
| SLEDAI, median (range) | | 9 (4-18) | N/A |
| Activity index (AI), median (range) | | 14 (6-24) | N/A |
| Chronic index (CI), median (range) | | 0.5 (0-8) | N/A |
| **Laboratory test (normal range), N, median (range)** | | | |
| ESR (0-20mm/h) | | 22.5 (2-76) | N/A |
| CRP (<6mg/L) | | 0.5 (0.4-1) | N/A |
| IgG (7-16g/L) | | 9.21 (3.41-24.7) | N/A |
| IgA (0.7-4g/L) | | 2.11 (1.26-3.79) | N/A |
| IgM (0.4-2.3g/L) | | 1.28 (0.22-2.08) | N/A |
| IgG4 (0.4-2.3g/L) | | 0.31 (0.08-0.75) | N/A |
| C3 (0.82-1.8g/L) | | 0.39 (0.16-1.08) | N/A |
| C4 (100-400mg/L) | | 42 (20-291) | N/A |
| WBC (4-10×10^9^/L) | | 5.95 (3.9-9.6) | N/A |
| Hb (female, 113-151, male, 131-172g/L) | | 109.5 (87-134) | N/A |
| PLT (100-300×10^9^/L) | | 158.5 (64-449) | N/A |
| eGFR (mL/(min×1.73m^2^) | | 89.33 (16.62-147.63) | N/A |
| SCr (40-88mmol/L) | | 57 (45-397) | N/A |
| BUN (2.8-7.6mmol/L) | | 7.19 (3.1-27.6) | N/A |
| UA (154-357μmol/L) | | 428 (186-671) | N/A |
| β2-microglobulin (1-3mg/L) | | 3.26 (2.89-33.21) | N/A |
| Serum cystatin C (<1.03mg/L) | | 1.67 (0.8-4.47) | N/A |
| Urinary NAG enzyme (<20U/g·Cr) | | 18.15 (7.1-47.67) | N/A |
| Urinary α1 microglobulin (<15mg/g·Cr) | | 22.55 (5.57-77.08) | N/A |
| Urinary IgG (<12mg/g·Cr) | | 67.17 (11.85-912.5) | N/A |
| Urinary transferrin (<2.9mg/g·Cr) | | 113.3 (5.49-660.84) | N/A |
| Urinary microalbumin (<25mg/g·Cr) | | 2325.7 (93.22-8531.5) | N/A |
| 24h urine protein quantification (28-141mg/24h) | | 2888.5 (196-6905) | N/A |
| **Medication, Positive rate, n (%)** | |  |  |
| Untreated (first visit) | | 1 (12.5) | N/A |
| Methylprednisolone | <40mg/day | 2 (25) | N/A |
|  | ≥40mg/day | 5 (62.5) | N/A |
| Immunosuppressants | Tacrolimus | 1 (12.5) | N/A |
|  | Mycophenolate Mofetil | 2 (25) | N/A |
|  | Cyclophosphamide | 1 (12.5) | N/A |
|  | Hydroxychloroquine | 4 (50) | N/A |

All the lab data from urine and serum were obtained at the time of kidney biopsy. Abbreviations: SLEDAI, systemic lupus erythematosus disease activity index; ESR, erythrocyte sedimentation rate; CRP, C-reactive protein; C3/C4, complement 3/4; WBC, white blood cell; Hb, hemoglobin; PLT, platelet; eGFR, Estimated glomerular filtration rate; SCr, serum creatinine; BUN, Blood urea nitrogen; UA, Uric Acid.
