# Supplementary figures and images for: Multi-omics profiling reveals epidermal growth factor as a potential biomarker and therapeutic target in lupus nephritis and ANCA-associated vasculitis with rapidly progressive glomerulonephritis
Source: PLoS One. 2026 May 29;21(5):e0349307. doi: 10.1371/journal.pone.0349307 (PMC13221000; doi:10.1371/journal.pone.0349307)

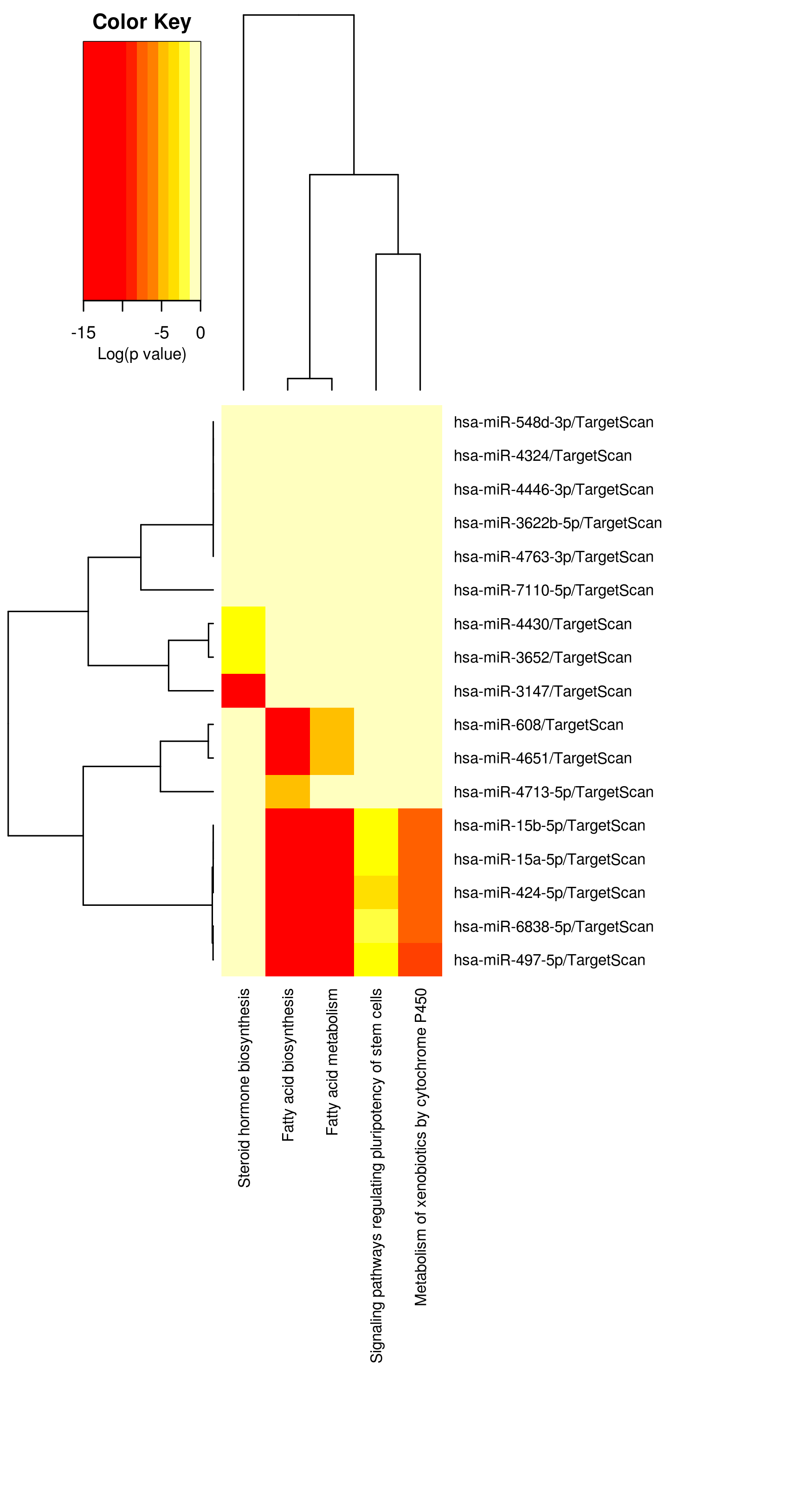

Supplement: S1 Fig — Pathway enrichment of 17 miRNAs by DIANA-miRPath v3. 0 tool. (TIF) [file pone.0349307.s002.tif]

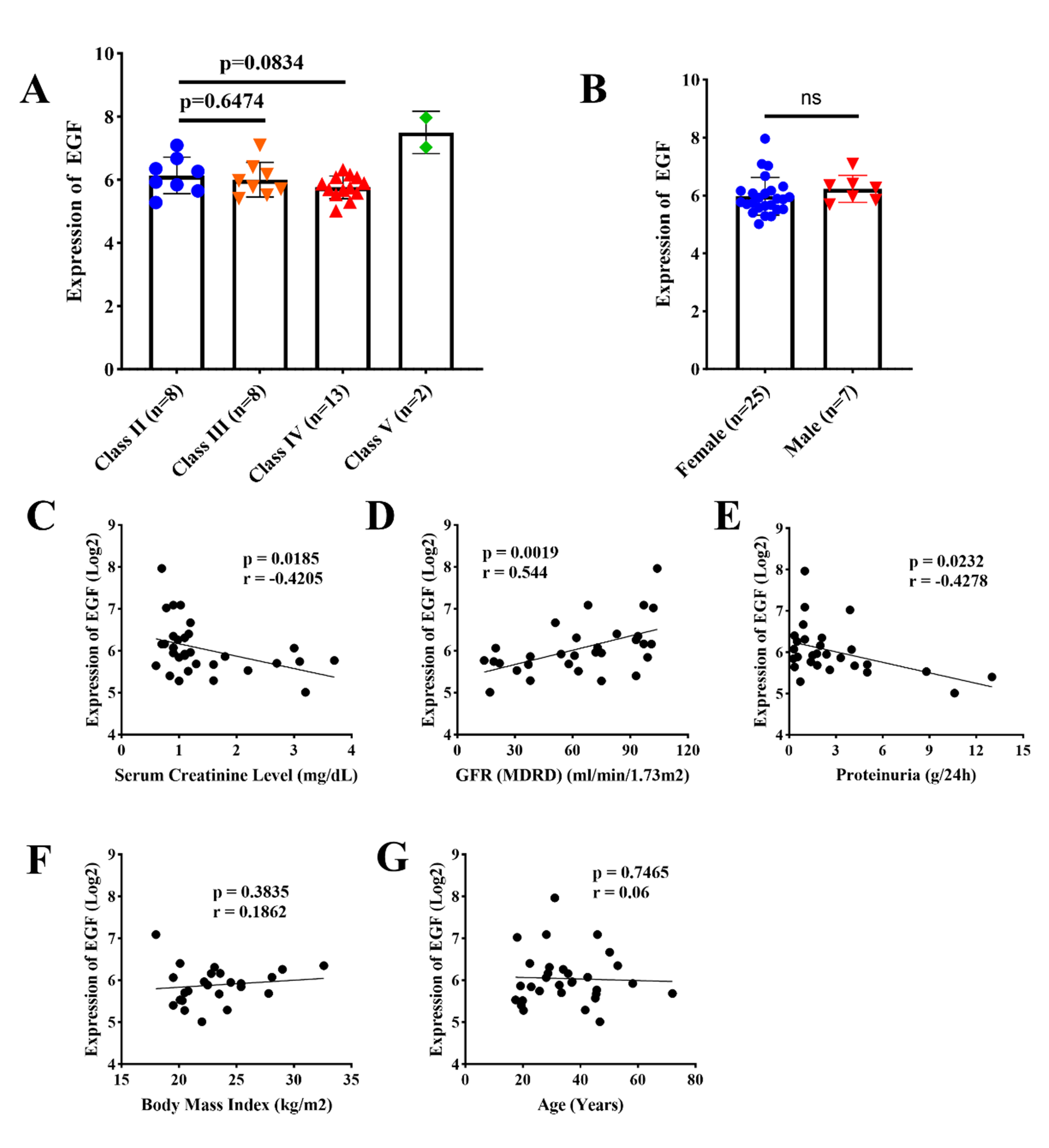

Supplement: S2 Fig — Relationship between hub genes and clinical characteristics in LN. A. Expression of EGF within the glomerular compartments in different classes of LN patients from GSE104948 cohort. B. Expression of EGF within the glomerular compartments in female and male LN patients from GSE104948 cohort. C-D. The correlation between expression of EGF and serum creatinine level (C), glomerular filtration rate (GFR) (D), proteinuria level (E), body mass index (F) and age (H) in LN patients. *, P < 0.05; **, P < 0.01; ***, P < 0.001; ns, no significant. P < 0.05 was considered statistically significant. (TIF) [file pone.0349307.s003.tif]

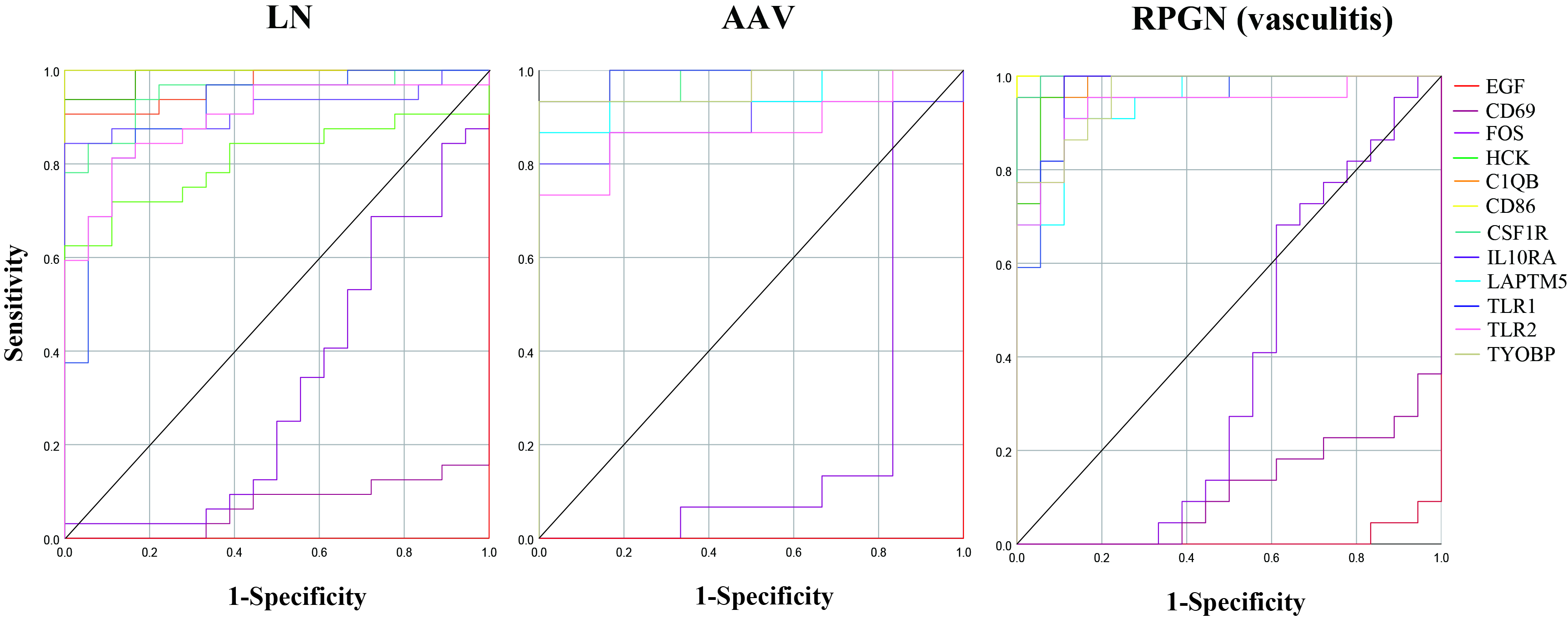

Supplement: S3 Fig — ROC curve of 12 hub genes in LN, AAV and RPGN (vasculitis). (TIF) [file pone.0349307.s004.tif]

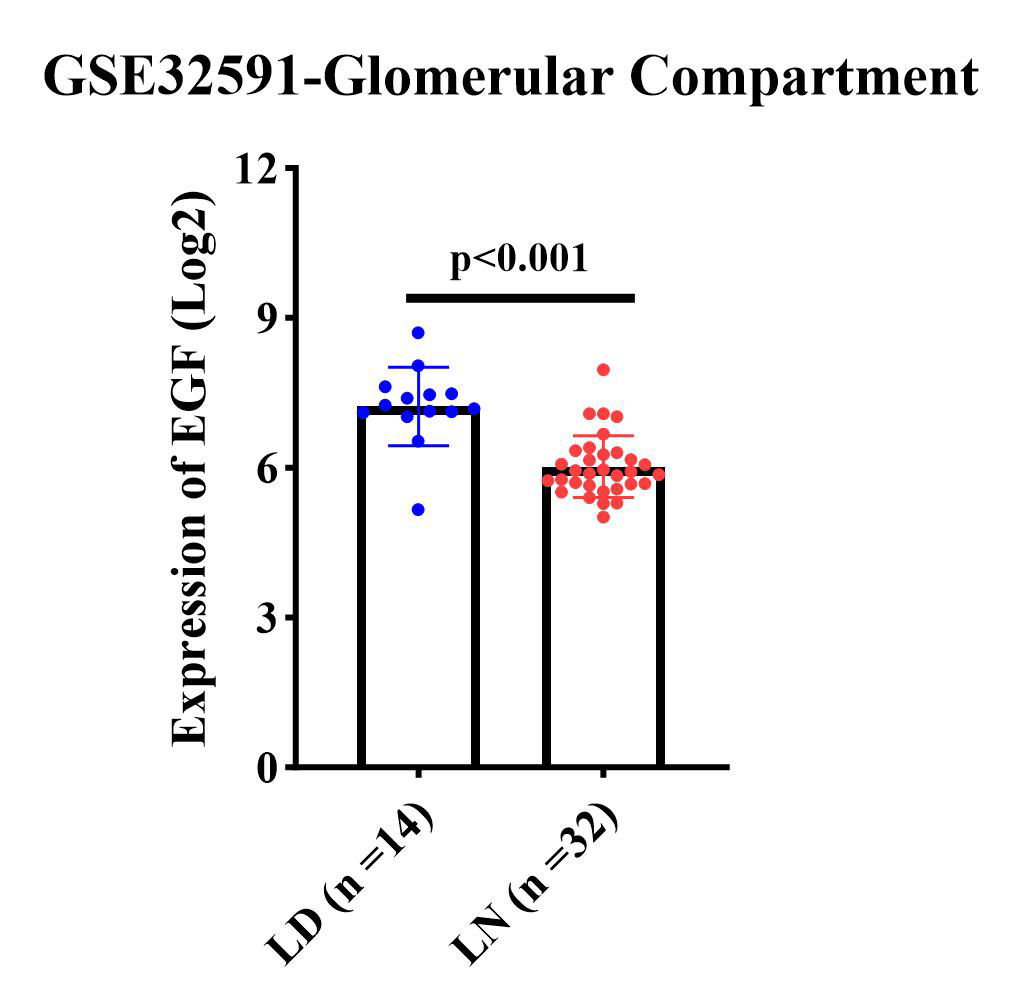

Supplement: S4 Fig — Expression of EGF in validation set (GSE32591 cohort). (TIF) [file pone.0349307.s005.tif]

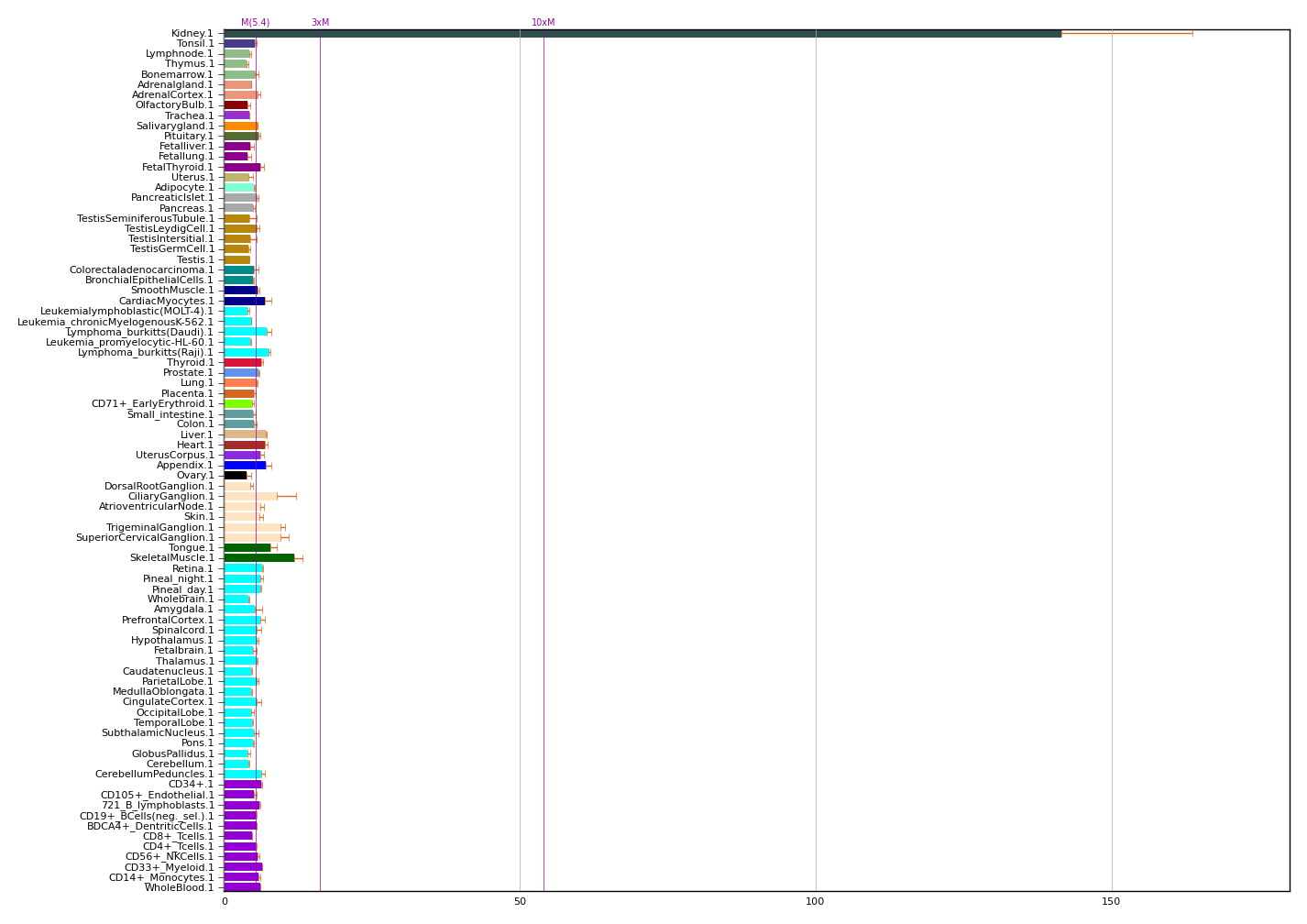

Supplement: S5 Fig — Expression of EGF in in different organs or tissues (BioGPS). (TIF) [file pone.0349307.s006.tif]
